# Nitrogen fertilizer rate increases plant uptake and soil availability of essential nutrients in continuous maize production in Kenya and Zimbabwe

**DOI:** 10.1007/s10705-019-10016-1

**Published:** 2019-09-07

**Authors:** Heather R. Pasley, Jill E. Cairns, James J. Camberato, Tony J. Vyn

**Affiliations:** 1 Agronomy Department, Purdue University, West Lafayette, IN, USA; 2 International Maize and Wheat Improvement Centre (CIMMYT), PO Box MP163, Harare, Zimbabwe

**Keywords:** Maize hybrids, Fertilizer, Nutrient depletion, Nutrient availability, Food security

## Abstract

Low fertilizer application rates for several decades have depleted soil nutrients in Sub-Saharan Africa (SSA) and contributed to relatively stagnant maize (*Zea mays* L.) yields. As maize is a staple crop, nutrient depletion has resulted in major food insecurity. While one potential solution is to apply more nitrogen (N) fertilizer, previous studies in SSA have found maize yield responses to be variable, likely because N is often not the only limiting nutrient. This study aimed to determine the impact of consecutive N fertilizer applications on plant uptake and available soil reserves of non-N nutrients. Maize was grown continuously in 3 sites that were representative of the ecosystem variability found in East/Southern Africa (Embu, Kenya; Kiboko, Kenya; Harare, Zimbabwe) at 4 different N fertilizer rates (0–160 kg N ha^−1^) from 2010 to 2015. Following the final season, grain, stover, and soil (sampled at different depths to 0.9 m) samples were analyzed for essential plant nutrients. Nitrogen fertilizer increased plant uptake of P, S, Cu, and Zn by up to 280%, 320%, 420%, and 210%, respectively, showing potential for mitigating non-N nutrient deficiencies in 2 of the 3 sites. Cumulatively, however, there was a net negative effect of higher N rates on the P, K, and S soil-plant balances in all sites and on the Mn and Cu soil-plant balance in Kiboko, indicating that applying N fertilizer depletes non-N soil nutrients. While N fertilizer enhances the uptake of non-N nutrients, a balanced application of multiple essential nutrients is needed to sustainably increase yields in SSA.

## Introduction

Sub-Saharan Africa (SSA) is facing a major food security crisis as crop yields fail to meet the needs of its growing population in part due to poor soil fertility (Denning et al. 2009; Lal 2009; Tully et al. 2015). Agriculture is the major source of income for more than 65% of the people in SSA (Oluoch-Kosura and Sikei 2013); however, land degradation and an inability to afford fertilizers for both production and remediation purposes has threatened many people with food insecurity (Bosede 2010; Oluoch-Kosura and Sikei 2013). Estimates have placed maize’s yield gap in SSA at 200% and have projected that the average yield needs to increase at an annual rate of 2% by 2050 in order to feed the growing population (Fischer et al. 2009; Cassman and Grassini 2013; Ten Berge et al. 2019). Overall, SSA demand for more cereals is disproportionally growing at approximately 5 times the global rate (Van Ittersum et al. 2016).

Maize is a primary source of grain in Eastern and Southern Africa where the average person (based on each country’s population) consumes up to 85 kg of maize per year (Smale et al. 2013). Globally, after water, nitrogen (N) is often the biggest limiting factor for cereal production. Increasing the amount of N fertilizer applied has frequently been suggested as a potential way to enhance yields (Mueller et al. 2012; Ten Berge et al. 2019). However, previous studies have found that the yield response to N fertilizer is inconsistent, likely because low N is not the only stress in the system (Sileshi et al. 2010).

Prior to evaluating the yield benefits of applying N fertilizer, it is necessary to determine what non-N nutrients are yield-limiting and how the application of N fertilizer impacts their availability (Kihara et al. 2016). As certain non-N nutrients tend to interact with each other, the availability of one may limit that of another (Marschner 2011; Bindraban et al. 2015; Tully et al. 2015). Therefore, it is important to look at the status of multiple nutrients at once. Globally, studies have looked at the impact of N fertilizer on the uptake of non-N nutrients such as phosphorus (P), potassium (K), and sulfur (S) (Feil et al. 1992; Holou et al. 2011; Ciampitti et al. 2013) and micronutrients (Ciampitti and Vyn 2013), but no such studies exist in SSA. These global studies have primarily focused on higher N fertilizer rates than what is typically applied in SSA, limiting the direct applicability of their findings to smallholder farmers in SSA. When the impact of applying N fertilizer on cropping systems has been studied in SSA, the focus is on yield response, not on changes in non-N nutrient uptake (Twomlow et al. 2010; van der Velde et al. 2013; Vanlauwe et al. 2015; Tovihoudji et al. 2017). Moreover, while previous work in SSA has found that soil manganese (Mn), copper (Cu), zinc (Zn), and P concentrations need to be investigated for potential deficiencies, rarely have these nutrients been looked at simultaneously in a field environment in SSA (Christianson and Vlek 1991; Mueller et al. 2012; Ezui et al. 2016; Marenya and Barrett 2017). With the growing interest in non-N nutrient fertilizer microdosing as a pathway for sustainable intensification (Twomlow et al. 2010), it is necessary to first look at the impact of applying N fertilizer on non-N nutrients as N currently is the most widely applied nutrient (van der Velde et al. 2014).

Grain yields in SSA are not only limited by low nutrient inputs, but also by drought stress and high erosion rates. Sporadic but intense rainfall events and high evapotranspiration rates make drought-like conditions and high erosion rates common in SSA cropping systems (Guuroh et al. 2018). In such drought-susceptible, highly-erodible locations, deeper rooting systems are advantageous and so it is important to look not only at the availability of the nutrients in the top soil, but also at deeper depths (Rao et al. 2016). While deeper roots are beneficial for sourcing water in dry conditions, they may impede the plant’s ability to access some nutrients, since nutrient concentrations often decrease with depth. Previous studies have taken only the top 0.1–0.2 m into account when assessing the fertility level of the soil (Ouédraogo et al. 2007; Zingore et al. 2007; Marenya and Barrett 2017). In contrast, this study investigated the top 0.9 m in an effort to encompass the majority of the rooting zone.

The objectives of this study were to estimate the long-term impact of N fertilizer on non-N nutrient depletion, quantify the soil non-N nutrient depletion at different N rates, and to determine whether the application of N fertilizer and/or stratification in the rooting profile impacts the availability and maize plant uptake of non-N nutrients.

## Materials and methods

### Site description

**Fig. 1:**
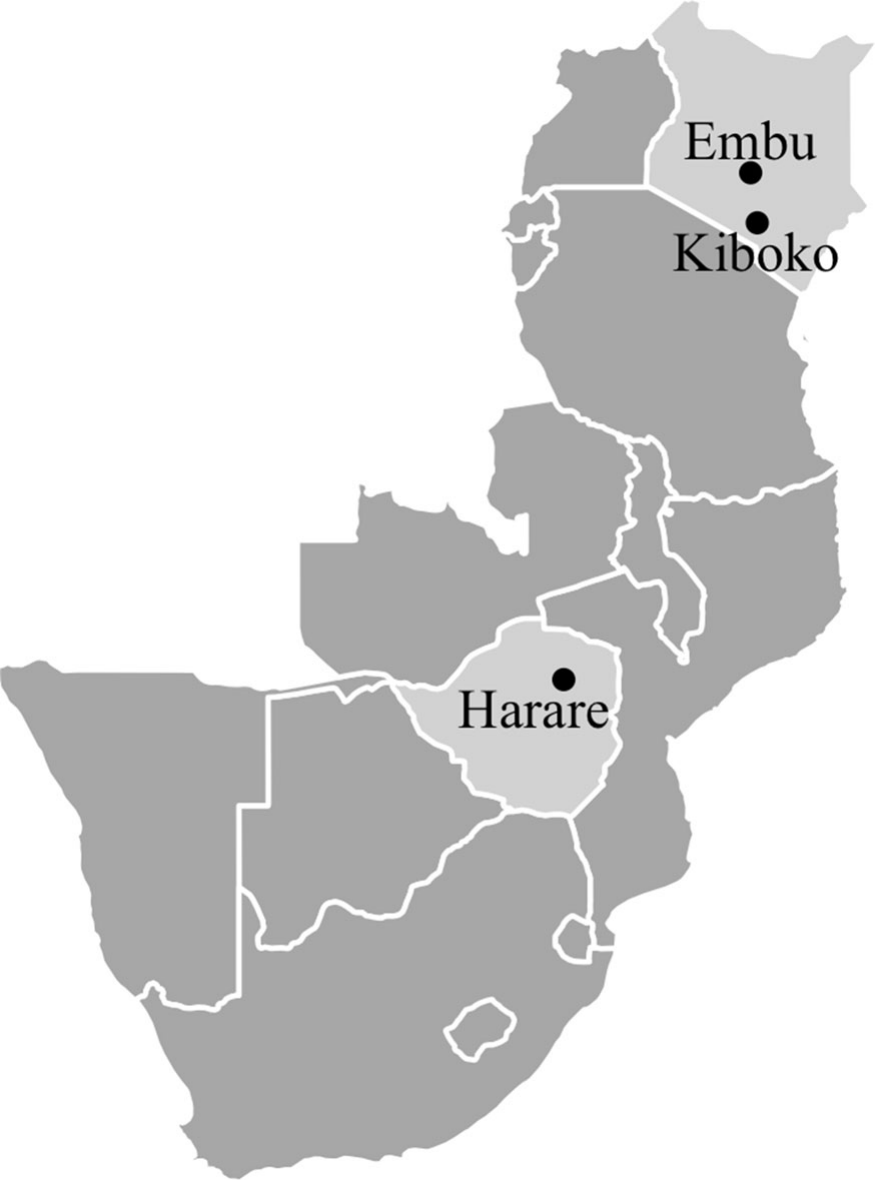
Experimental site locations

Three field experiments (Embu, Kenya (00°31′S 37°29′E); Kiboko, Kenya (02°13′S 37°42′E); Harare, Zimbabwe (17°43′S 31°5′E)) (Fig. 1) have been under continuous maize cultivation since 2010. There were two growing seasons planted each year in the Kenya sites (one short rains (SR) season (Embu: October–March; Kiboko: December–April) and one long rains (LR) season (Embu: April–September; Kiboko: May–October)) and one growing season a year in Zimbabwe (December–July). In total, there were 9 seasons in Embu, 7 in Kiboko, and 5 in Zimbabwe.

Embu is located in the temperate highlands of Kenya, Kiboko in the dry mid-altitude zone of Kenya, and Harare in the wet upper mid-altitude zone of Zimbabwe (Cairns et al. 2013). Soils were Humic Nitisols in Embu, Acri-Rhodic Ferrassols in Kiboko, and Ferric Luvisols in Harare (Table 1).

**Table 1:** Soil characteristics of Embu, Kiboko, and Harare: FAO soil classification, texture, bulk density (BD), %Organic Matter (OM), C:N, amorphous Fe, cation exchange capacity (CEC), pH (1:1 soil water slurry), and exchangeable acidity (for Embu and Harare)

Depth (m)	FAO soil classification	Texture	BD (Mg m^−3^)	OM (%)	C:N	Amorph. Fe (mg kg^−1^)	CEC (meq 100 g^−1^)	pH	Exch acidity (meq 100 g^−1^)
Embu									
0–0.15	Humic Nitisol	Clay loam	0.93	5.79	11	16.32	11.89	5.15	3.51
0.15–0.3		Clay	1.03	5.52	11	16.26	12.35	5.12	3.51
0.3–0.45		Clay	0.95	5.15	12	15.99	12.59	5.24	3.51
0.45–0.6		Clay loam	0.94	4.00	11	15.99	10.00	5.42	3.51
0.6–0.9		Clay loam	0.92	3.21	11	19.32	8.18	5.46	1.75
Kiboko									
0–0.15	Acri-Rhodic Ferrasols	Sandy loam	1.53	2.61	12	5.35	9.44	7.82	
0.15–0.3		Sandy clay loam	1.49	2.28	12	5.36	7.57	7.63	
0.3–0.45		Sandy loam	1.42	2.08	12	4.93	8.11	7.64	
0.45–0.6		Sandy loam	1.47	1.70	11	5.40	6.47	7.28	
0.6–0.9		Sandy clay loam	1.49	1.53	12	5.29	6.66	7.07	
Harare									
0–0.15	Ferric Luvisols	Clay loam	1.37	2.59	12	13.38	7.82	5.59	2.85
0.15–0.3		Clay	1.38	1.95	12	14.49	8.48	5.73	1.75
0.3–0.45		Clay	1.32	2.05	12	12.78	9.52	5.82	2.63
0.45–0.6		Clay	1.30	1.36	12	12.09	7.51	5.88	1.32
0.6–0.9		Clay	1.34	1.42	12	9.75	8.21	5.9	1.46

Rainfall and temperature data (NASA 2017) is listed in Supplementary Table 1. Rainfall distribution indices were used to quantify the risk of drought or heat stress in the sites (Bronikowski and Webb 1996; Tremblay et al. 2012; Supplementary Table 1). Embu’s low rainfall levels in most seasons and low distribution of rainfall during the critical period (the window of time starting 2 weeks before anthesis and ending 2 weeks after silking) and the grain filling period (estimated to have begun 2 weeks after silking and to end at harvest) indicated a high risk of drought stress. Unlike Embu, rainfall in the Kiboko and Harare sites were supplemented by irrigation in order to mitigate any potential drought stress.

### Treatment, experimental design, and field management

Management details are listed in Supplementary Table 2. The experiment was a split-plot design with four replications in which N rate (0, 30, 60, and 90 kg N ha^−1^ in Embu; 0, 40, 80, and 160 kg N ha^−1^ in Kiboko and Harare) was the main plot and hybrid, the sub-plot. Six maize hybrids were selected for each site based on results from low N and optimal N screenings for grain yield and/or popularity in the targeted country (Supplementary Table 3; Fisher et al. 2015). The locations of the treatments were randomized the first season, but were fixed thereafter. Two hybrid treatments were intentionally duplicated in two locations to provide comparative data from which the hybrids’ performance stability across different environments might be inferred. The hybrid Duma43 was planted at both Embu and Harare and the hybrid H513 was planted at both Embu and Kiboko.

**Table 2:** Reference concentration ranges for N, P, K, S, Mn, Zn, and Cu in the upper 0.2 m of soil

Nutrient	Concentration range (mg kg-1)	Location	Reference
N	360–1840; 600–5000	West Africa; USA	Vanlauwe et al. (2000) (Kjeldahl); Bremner and Mulvaney (1982) (Kjeldahl)
P	1–53; 4.6–13.2; 40–60	Global (highly weathered soils); West Africa; Midwest, USA	Sharpley et al. (1987) (Mehlich 1); Vanlauwe et al. (2000) (Olsen); A&L Great Lakes Lab (2001) (Bray)
K	94–180; 105–156	West Africa; Midwest, USA	Sparks 2012 (NH4OAc)
S	3–22; 1.8–7.5	Ghana; Iowa, USA	Acquaye and Kang (1987) (KH2PO^−^_4_); Neptune et al. (1975) (LiCl)
Mn	4–140; 14–22	Global; Midwest, USA	Sillanpaa (1982); A&L Great Lakes Laboratories (2010) (Mehlich 3)
Zn	0.62–6.03; 0.2–5.2; 1.8–2.5	South Africa/Zimbabwe; Malawi; Midwest, USA	Herselman et al. (2005) (EDTA); Nyamangara and Mzezewa (1999) (EDTA); Snapp (1998) (EDTA); A&L Great Lakes Laboratories (2010) (Mehlich 3)
Cu	0.84–10.6; 1–1.8	South Africa/Zimbabwe; Midwest, USA	Herselman et al. (2005) (EDTA); Nyamangara and Mzezewa (1999) (EDTA); A&L Great Lakes Laboratories (2010) (Mehlich 3)

Values were sourced from studies on African maize cropping systems where available. In this paper, values termed as “low,” “within the normal range,” or “high” according to if they were below, within, or above these ranges, respectively

**Table 3:** Soil Mehlich 3 extractable macro/micro-nutrient concentrations for each depth averaged over all hybrids and N rates in the Embu 2015SR season, Harare 2014/15 season, and Kiboko 2015SR season post-harvest

		Embu						Harare						Kiboko					
		P mg kg^−1^	K	S	Mn	Zn	Cu	P mg kg^−1^	K	S	Mn	Zn	Cu	P mg kg^−1^	K	S	Mn	Zn	Cu
Depth (m)	0–0.15	24 a	373 a	22 c	279 a	16 a	1.2 a	21 a	135 a	28 c	138	13 a	10.2	107 a	410 a	15 d	75 a	3.2 a	3.7 b
	0.15–0.3	8 b	238 b	25 b	259 b	14 b	1.2 a	4 b	62 b	33 b	126	11 ab	9.6	52 b	348 be	23 c	63 b	1.0 c	4.0 a
	0.3–0.45	3 c	226 b	25 b	237 c	12 c	1.1 b	3 b	59 b	34 ab	125	10 b	8.6	62 b	386 b	26 c	62 b	2.4 b	3.7 b
	0.45–0.6	< 1 d	131 c	28 b	179 d	8 d	0.8 c	< 1	c 31 d	35 ab	126	8 c	6.8	23 d	336 c	54 b	51 c	1.1 c	3.6 c
	0.6–0.9	< 1 d	131 c	37 a	159 d	7 e	0.7 d	< 1	c 38 c	44 a	118	8 c	5.9	33 c	333 c	84 a	53 c	1.5 c	3.4 d
Effect	df	Level of significance						Level of significance						Level of significance					
N	3	ns	ns	ns	***	ns	ns	ns	**	**	ns	ns	ns	*	ns	ns	ns	*	ns
H(C)	4	ns	ns	ns	***	ns	ns	ns	ns	ns	ns	ns	***	ns	ns	ns	ns	ns	ns
C	1	ns	ns	ns	ns	*	ns	ns	***	ns	ns	ns	ns	ns	ns	ns	ns	ns	ns
D	4	**	***	*	***	***	***	***	***	*	ns	***	***	***	***	***	***	***	***

N is N rate, H(C) is hybrid, C is hybrid category, and D is Depth. Letters by values denote significant differences among the hybrids in a site (*p* < 0.05). “ns” denotes no significant difference (p > 0.05). *, **, *** denotes significance at p < 0.05, p < 0.001, and p < 0.0001, respectively. Interaction effects were not significant

Sub-plots at all sites were six 5 m long rows with 0.75 m spacing between rows and within-row spacing of 0.25 m in Embu, 0.2 m in Kiboko, and 0.3 m in Harare. Plots were hand-tilled immediately prior to planting and hand-planted with 2 seeds per hill. The plots were thinned by hand to one plant per hill at the desired population 4 weeks after planting. At all sites, 30% of the total N rate was broadcast applied at planting. The remaining 70% was broadcast applied at 5–6 weeks after planting, along with 20 kg P ha^−1^. In Kiboko, 1/3 of the stover biomass was returned postharvest. In Embu and Harare, all biomass was removed from the field at each harvest. In the 5 years prior to establishment of these experimental trials, a degree of soil N depletion was achieved using maize cultivation without N in Embu and Harare and using sorghum (*Sorghum bicolor* (L.)) cultivation in Kiboko.

The experiments in Embu were rainfed. Supplemental sprinkler irrigation was applied in Kiboko and Harare when the top 100 mm of soil felt dry.

### Field measurements and sampling

Each season, for grain yield determination, the grain was hand-harvested in the center 13.5 m^2^ area in each plot in Embu and Harare and the center 7.5 m^2^ area in each plot in Kiboko, leaving at least 1–2 plants as border at row ends. Differences in the sampling sizes among sites reflect differences in the overall plot sizes. Immediately prior to the harvest of 2015SR season in Embu, 2013LR and 2014SR seasons in Kiboko (Kiboko was not planted in 2015SR due to delays in seed acquisition), and of 2014/15 season in Harare, 6–10 whole plants were removed from the center of each plot and partitioned as grain and stover for whole plant nutrient analysis. Samples were ground to 1 mm and sub-sampled further before being shipped to Purdue University in Indiana for analyses. At Purdue, the plant samples were further ground to 100 µm diameter using a Retsch SS MM200 Ball Mill. Plant samples were analyzed for total C and N by the combustion method (Etheridge et al. 1998) using a Flash 2000 CHN Analyzer (ThermoFisher Scientific Inc.) and for total P, K, S, magnesium (Mg), calcium (Ca), Mn, Cu, and Zn using nitric acid digestion (Pequerul et al. 1993).

After harvest in 2015 at all sites, composite soil samples comprised of 5 or 15 soil cores per plot (5 in Embu due to excessively compacted nature of the soil) were taken to a depth of 0.9 m in 5 depth increments (0–0.15 m, 0.15–0.3 m, 0.3–0.45 m, 0.45–0.6 m, and 0.6–0.9 m). Soil samples were hand-ground, sieved through a 2 mm screen, and sub-sampled before analysis in the same laboratory that was mentioned above. Bulk density was measured using an intact core method (Blake 1965). The cores were 60 mm in height and 85 mm in diameter and 3 core samples were taken at each depth interval in each replication.

Soil texture was analyzed using the hydrometer method (Bouyoucos 1962). Soil pH was analyzed in a 1:1 soil slurry (McLean 1982) using an AR20 pH meter (ThermoFisher Scientific Inc.). Buffer pH and exchangeable acidity of acidic soils were measured using a single extraction with BaCl_2_ (Rhoades 1982; Schwertfeger and Hendershot 2009). Soil extracts were analyzed on an ICP-MS for Mehlich 3 extractable P, K, S, Mg, Ca, Mn, Cu, and Zn (Mehlich 1984). Mehlich 3 extraction was selected based on its capacity to measure multiple elements at once and its popular usage in the US, allowing for easy comparability to literature data. Exchangeable Al was extracted with 0.01 M CaCl_2_ and analyzed on an ICP-MS (Hoyt and Nyborg 1971; Bertsch and Bloom 1996). Amorphous Fe was analyzed using Tamm’s Reagent extraction in darkness (Loeppert and Inskeep 1996). A sub-sample was ground to 100 im diameter using a Retsch SS MM200 Ball Mill and analyzed for total C and N using the combustion method (Bremner and Mulvaney 1982; Nelson and Sommers 1982). Given the lack of carbonates in the soil, organic matter was estimated using the total C concentration and a widely accepted single factor estimation of organic matter to C conversion factor of 2:1 (Pribyl 2010).

All data discussed in this manuscript are averaged over all hybrids except where there is a significant N Rate × Hybrid interaction effect. Individual hybrid data and N dynamics of the overall study are discussed elsewhere (Pasley 2018). The results in this paper primarily refers to data collected at the harvest (plant) or post-harvest (soil) of Embu’s 2015SR season, Kiboko’s 2013LR season (for plant data) and 2015SR season (for soil data), and Harare’s 2014/15 season. Grain yield data, however, are shown as it was recorded for all seasons except for Kiboko’s 2012LR. Stover biomass was only measured in 2015SR season in Embu, 2013LR and 2014SR seasons in Kiboko, and 2014/15 season in Harare (Supplementary Table 4). The harvest indices listed in Supplementary Table 4 were used to estimate stover biomass for the other seasons from the grain yield data in order to calculate the cumulative total plant nutrient removal.

The degree to which soils were, on average, low or sufficient in regards to their non-N nutrient status was determined based on comparisons of the average soil nutrient concentrations with the typical concentration ranges found in tropical and temperate soils sourced from literature (Table 2). Average measured concentrations which fell below or above the previously reported ranges were considered *low* or *high*, respectively.

### Statistical analysis

Data was analyzed using SAS 9.4 PROC Mixed ANOVA and differences in Least Square Means were considered significant at α = 0.05. All differences and trends described in this paper were found to be significant unless otherwise noted. Blocks were random effects; all other factors were fixed. For plant data, seasons could not be pooled due to significant variance in residuals. A 2-tailed LSD (α = 0.05) was used to compare the resulting Least Squared Means to a constant. Three treatment factors were investigated: N rate, hybrid, and depth (for soil).

Quadratic plateau models were fit to the grain yield response to N rate averaged over all hybrids in order to calculate the agronomic optimum N rate (AONR) and the grain and stover nutrient concentrations at the AONR (Frank et al. 1990). This model was found to provide a superior fit for the data to quadratic and linear models (*P* < 0.05).

Single-degree of freedom contrasts (α = 0.05) were conducted on the data for the shared hybrids (Duma43 and H513) at comparable N rates in order to determine the impact of the environmental differences.

Pearson correlations were conducted to analyze the linear relationship between total soil P in the top 0.9 m and cumulative grain yield for all seasons (2011SR-2015SR in Embu; 2011LR-2014LR with the exception of 2012LR, where data was missing, in Kiboko; and 2013/14 and 2014/15 in Harare).

## Results

### Soil characteristics

Soil pH differed among the sites: pH was lower in Embu than in Harare and pH was much lower in both of these sites than in Kiboko (*P* < 0.0001; Table 1). Amorphous Fe levels were high in both Embu and Harare, but not in Kiboko which had pH > 7 at all depths.

There was less OM in Harare than in Embu (*P* < 0.0001), but no difference in OM between Embu and Kiboko (*P* = 0.06). In Kiboko, averaged across all depths, soil OM was higher at 80 kg N ha^−1^ (1.80%) than at the other N rates (1.52–1.57%) (*P* < 0.05). In Embu and Harare, soil OM did not differ among N rates. In all sites, soil OM decreased as depth increased (Table 1, *P* < 0.0001).

Soil Ca and Mg levels were consistent with the concentration range found in soils globally (950–1585 mg kg^−1^ Ca; 215–365 mg kg^−1^ Mg) (Camberato and Pan 2012). While there was a minor N rate effect on both Ca and Mg soil concentrations in Kiboko (not in the other sites), it likely resulted from either the irrigation water (which was high in both Ca and Mg) or the Ca in the calcium ammonium nitrate fertilizer applied and these differences, therefore, will not be discussed in this paper. There was a minor depth effect on both Ca and Mg soil concentrations in all three sites (data not shown). Neither were found to be yield-limiting factors in maize plants in any site (data not shown).

In the plots where the same hybrid (H513) was planted in both Embu and Kiboko, there was on average less soil P, K, S, Zn, and Cu but more soil Mn in Embu than in Kiboko. Average soil P, S, Cu, and Zn contents in Harare were lower than in Embu. The shared hybrid Duma43 extracted more Cu and less Zn in Harare than in Embu, but similar quantities of P and S.

### Agronomic optimal nitrogen rates

**Fig. 2:**
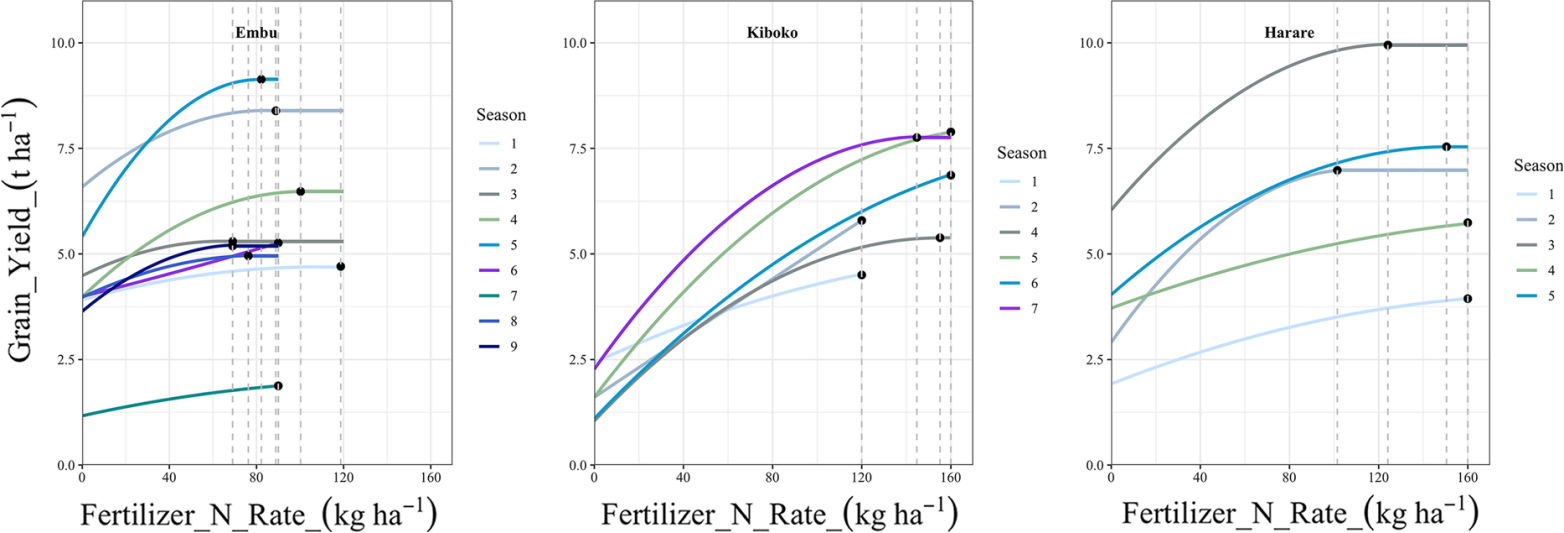
Grain yield (reported at 15.5% moisture) response to N rate averaged across all hybrids each season at Embu, Kiboko, and Harare. Points and dotted lines demark calculated agronomic optimal N rate (AONR). Where the AONR did not fall within the applied N rate range, the highest applied N rate was estimated to be the AONR

Even though the top N rates applied in the 3 sites far exceeded the average application rate of N fertilizer in SSA, the calculated agronomic optimal N rate (AONR) did not fall within the range of applied N rates in 3 out of 9 seasons in Embu, 4 out of 6 seasons in Kiboko, and 2 out of 5 seasons in Harare (Fig. 2).

### Macronutrients

**Fig. 3:**
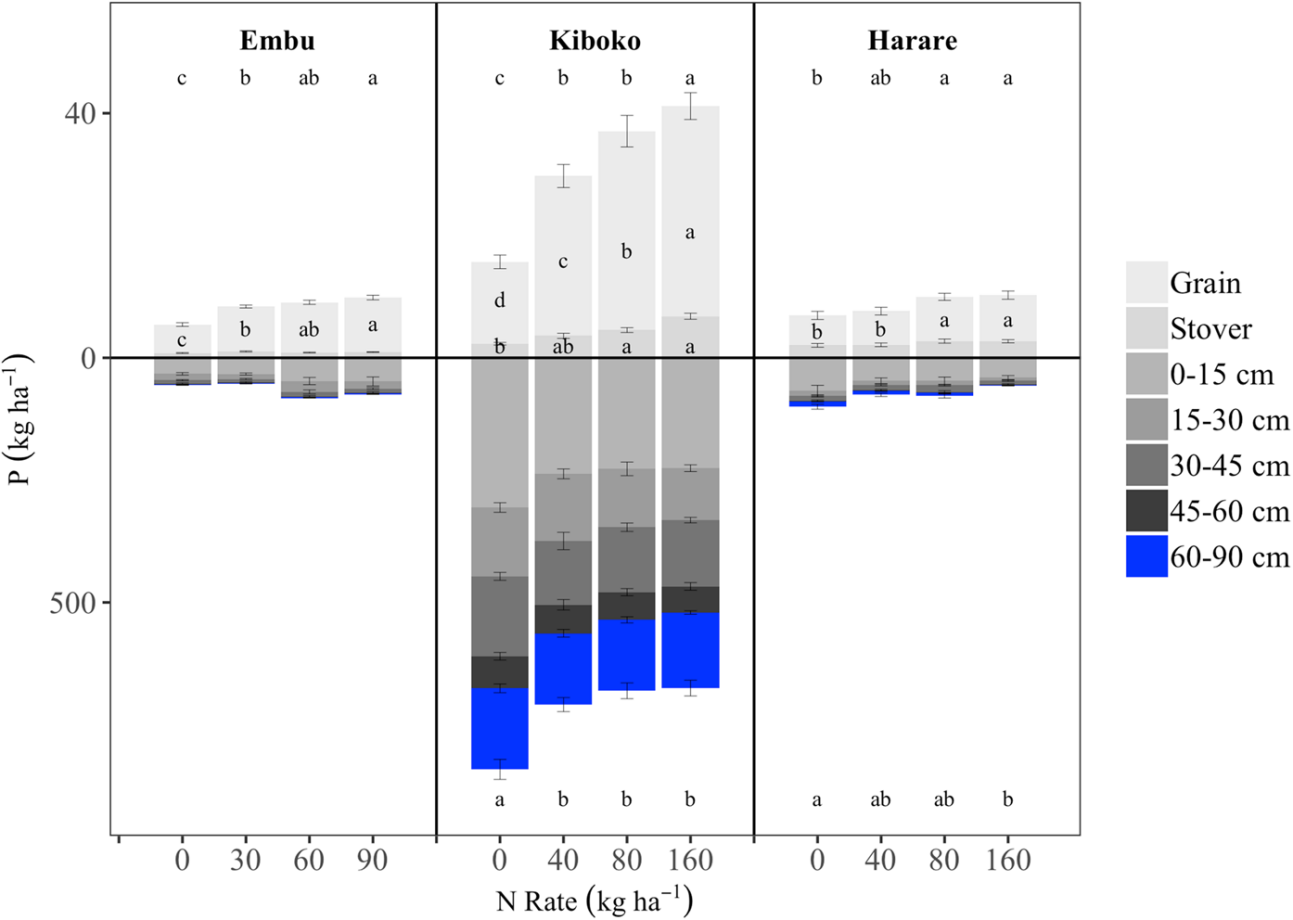
Phosphorus (P) plant-soil balance, averaged across all hybrids, in response to long-term N rates applied to continuous maize at Embu, Kiboko, and Harare. Letters denote differences among the hybrids’ total plant (above bars), grain/stover (on respective parts of bars), or total soil contents (below bars) (*p* ≤ 0.05). Where there are no letters, the difference was not significant

In Embu, total plant and grain P contents increased as the N rate increased (Fig. 3), but there was no N rate effect on inorganic soil P content in the top 0.9 m (Fig. 3). Inorganic soil P concentration decreased as depth increased from 24 mg kg^−1^ in the upper 0.15 m to < 1 mg kg^−1^ below a depth of 0.45 m (Table 3). In Kiboko, the total plant, grain, and stover P contents increased as the N rate increased, although stover P content plateaued at 80 kg N ha^−1^ (Fig. 3). There was less total inorganic soil P content in the top 0.9 m at the non-zero N rates (40, 80, and 160 kg N ha^−1^) than at 0 kg N ha^−1^ (Fig. 3). Inorganic soil P concentration was very high (107 mg kg^−1^) in the top 0.15 m and well above normal down to 0.45 m (Table 2), but declined to around 30 mg kg^−1^ below a depth of 0.45 m (Table 3). Inorganic soil P levels were at least 5 times higher in all depth increments at Kiboko than at Embu and Harare (Table 3). In Harare, total plant, grain, and stover P contents increased as the N rate increased and plateaued at 80 kg N ha^−1^ (Fig. 3). Nevertheless, there was no N rate effect on the total inorganic soil P content in the top 0.9 m (Fig. 3). Inorganic soil P concentration decreased as depth increased from 21 mg kg^−1^ in the top 0.15 m to < 1 mg kg^−1^ below a depth of 0.45 m (Table 3).

**Fig. 4:**
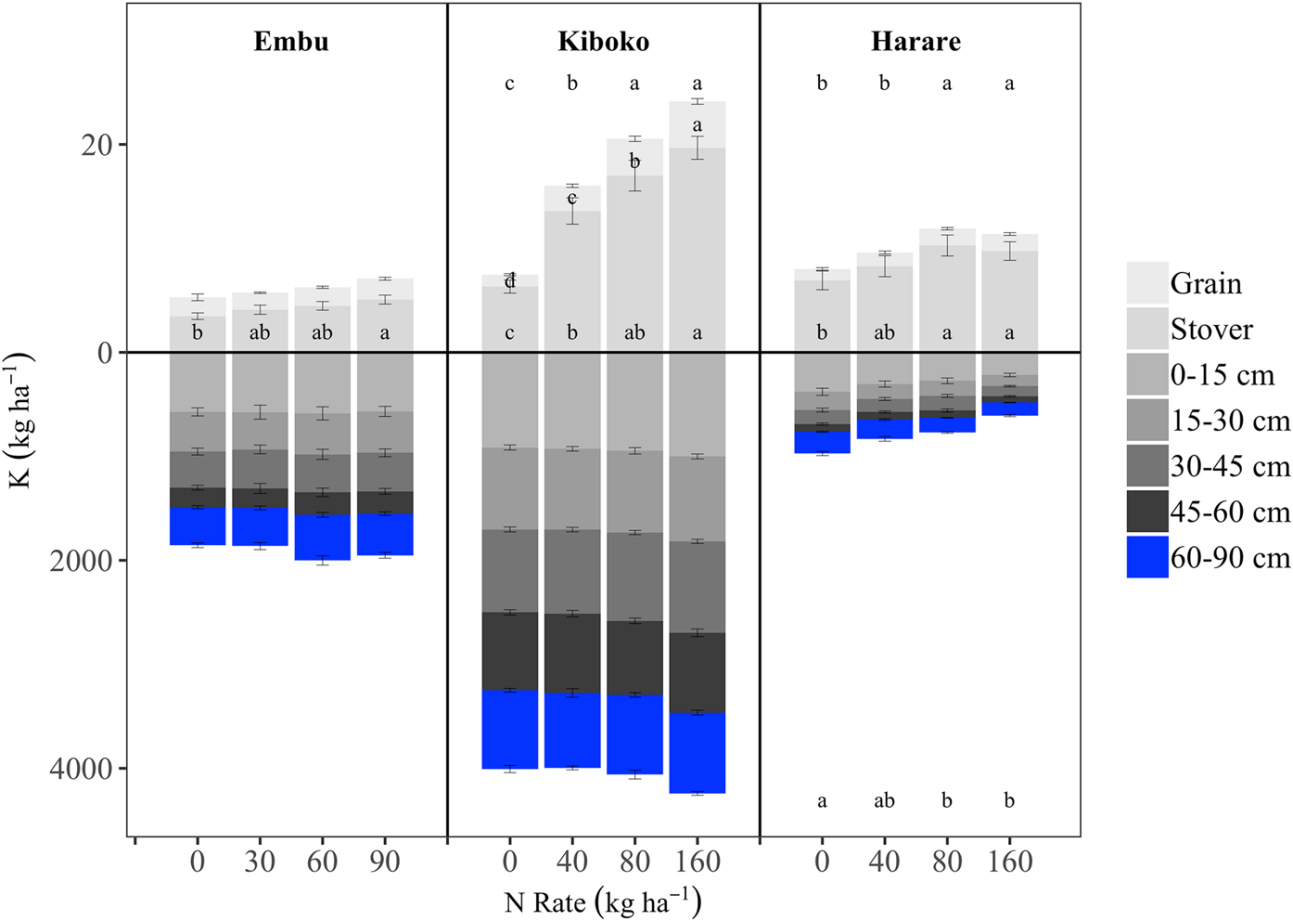
Potassium (K) plant-soil balance, averaged across all hybrids, in response to long-term N rates applied to continuous maize at Embu, Kiboko, and Harare. Letters denote differences among the hybrids’ total plant (above bars), grain/stover (on respective parts of bars), or total soil contents (below bars) (*p* ≤ 0.05). Where there are no letters, the difference was not significant

In Embu, there was no N rate effect on total plant, grain, or exchangeable soil K contents (Fig. 4). Stover K content increased as the N rate increased and plateaued at 30 kg N ha^−1^ (Fig. 4). The exchangeable soil K concentration in the top 0.15 m (373 mg kg^−^ ) was higher than the values considered normal in literature, and were still in the optimal range at the lower depths (131–238 mg kg^−1^) (Table 3). In Kiboko, total plant, grain, and stover K contents increased as N rate increased (Fig. 4). There was no N rate effect on total exchangeable soil K content in the top 0.9 m (Fig. 4). Even though exchangeable soil K concentrations were lower at the deeper depths than at the 0–0.15 m depth interval, they were high (333–410 mg kg^−1^) at all depths (Table 3). In Harare, there was no N rate effect on grain K content, but the total plant and stover K content increased as N increased and plateaued at 40 kg N ha^−^ (Fig. 4). Total exchangeable soil K content in the top 0.9 m decreased as the N rate increased (Fig. 4). The exchangeable soil K concentration decreased from 135 mg kg^−1^ in the top 0.15 m to around 35 mg kg^−1^ at depths below 0.45 m (Table 3).

**Fig. 5:**
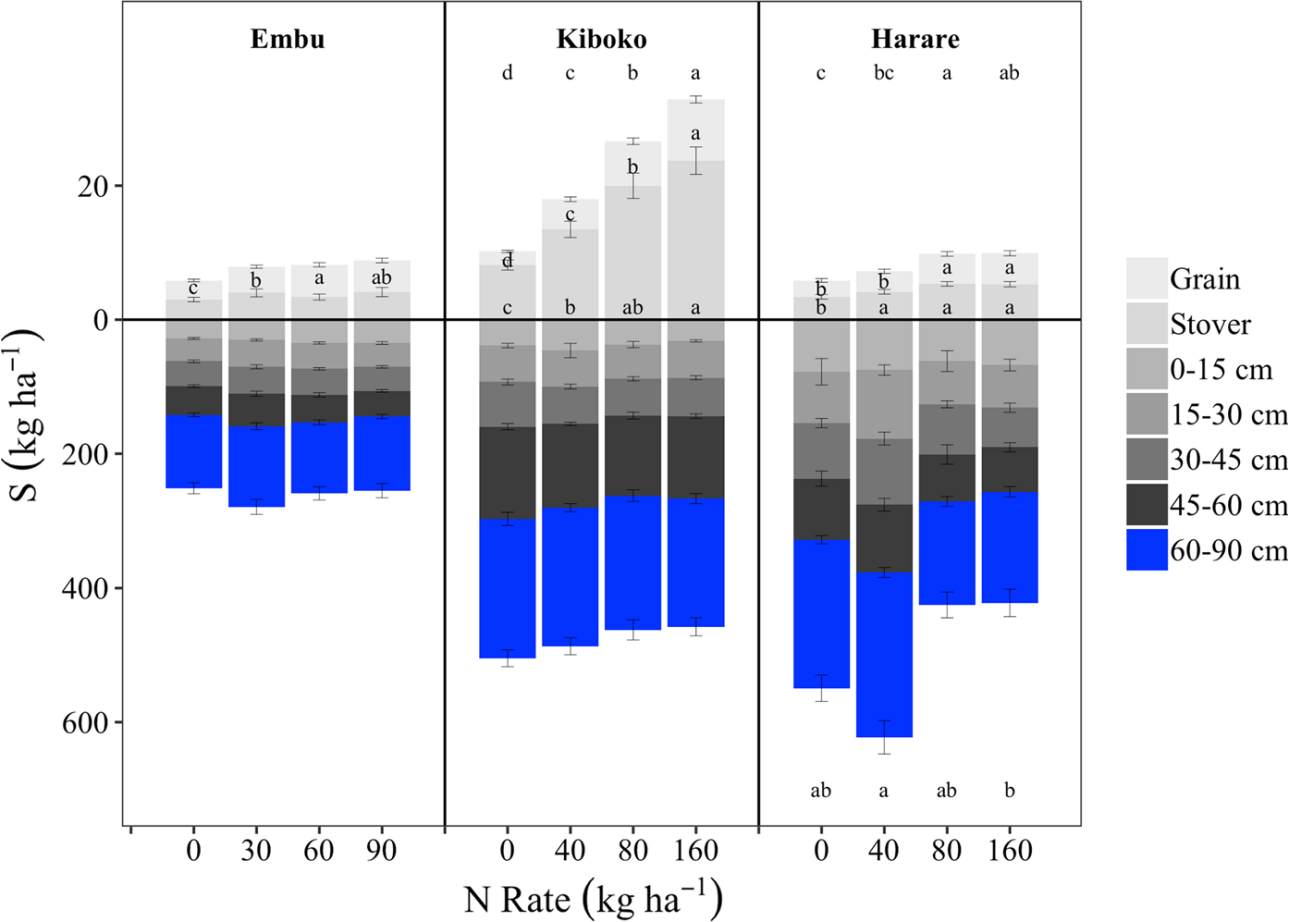
Sulfur (S) plant-soil balance, averaged across all hybrids, in response to long-term N rates applied to continuous maize at Embu, Kiboko, and Harare. Letters denote differences among the hybrids’ total plant (above bars), grain/stover (on respective parts of bars), or total soil contents (below bars) (*p* ≤ 0.05). Where there are no letters, the difference was not significant

In Embu, total plant and grain S uptakes were higher at the non-zero N rates (30, 60, and 90 kg N ha^−1^) than at 0 kg N ha^−1^ (Fig. 5). There was no N rate effect, however, on the total inorganic soil S content in the top 0.9 m (Fig. 5). In Kiboko, total plant, grain, and stover S uptakes increased as the N rate increased (Fig. 5). There was no significant N rate effect on the total inorganic soil S content in the top 0.9 m. In Harare, the total plant, grain, and stover S uptakes at 80 and 160 kg N ha^−1^ were higher than the comparable uptakes at 0 or 40 kg N ha^−1^ (Fig. 5). Total inorganic soil S content in the top 0.9 m was greater at 40 kg N ha^−1^ than at the other N rates (Fig. 5). Average inorganic soil S concentrations at each depth increment were high in each location (ranging from 22 to 37 mg kg^−^ at Embu, 15–84 mg kg^−1^ at Kiboko and 28–44 mg kg^−1^ at Harare; Table 2) and consistently increased as depth increased (Table 3).

### Micronutrients

**Fig. 6:**
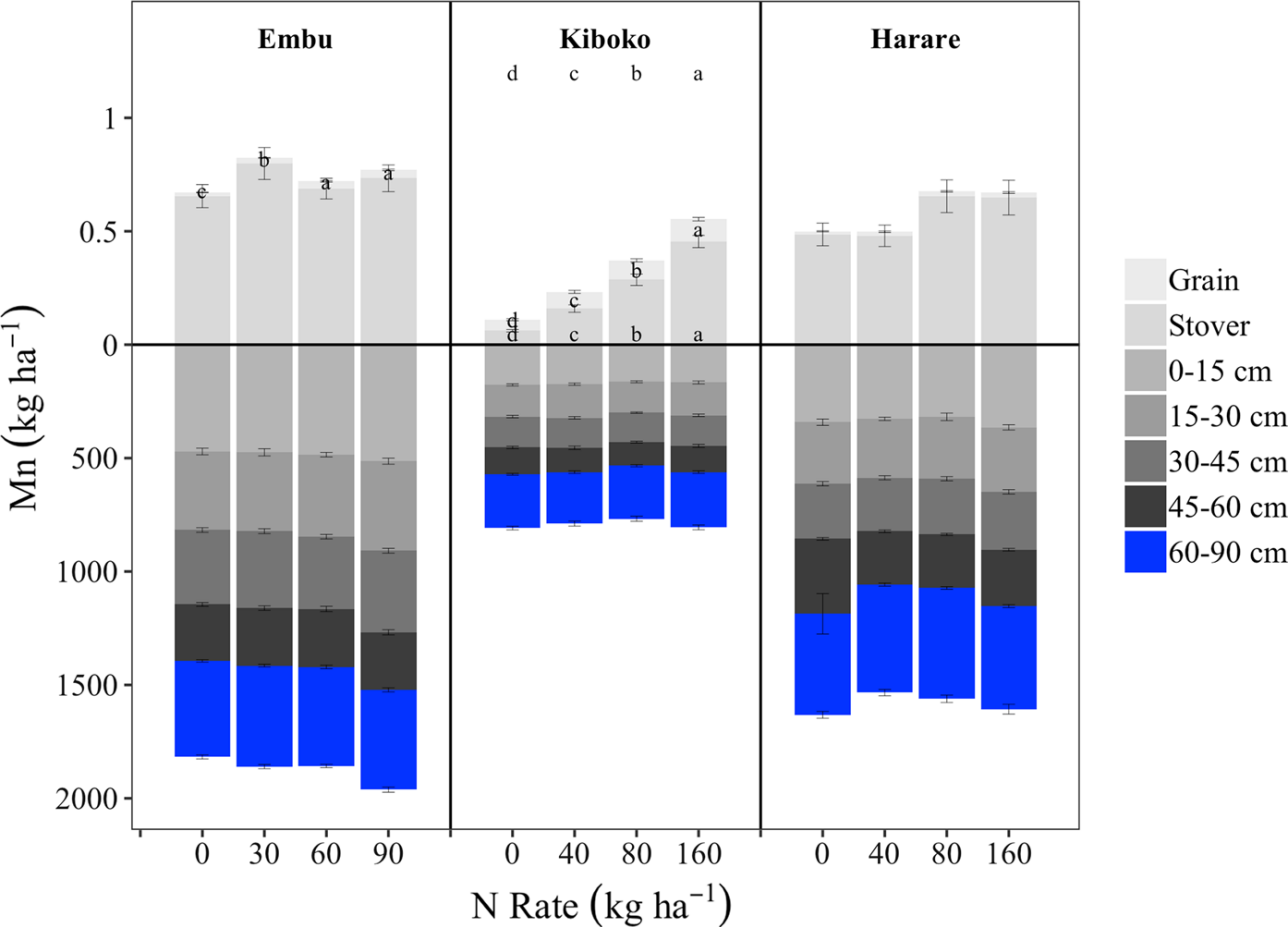
Manganese (Mn) plant-soil balance, averaged across all hybrids, in response to long-term N rates applied to continuous maize at Embu, Kiboko, and Harare. Letters denote differences among the hybrids’ total plant (above bars), grain/stover (on respective parts of bars), or total soil contents (below bars) (*p* ≤ 0.05). Where there are no letters, the difference was not significant

In Embu, total plant and grain Mn uptake was higher at 30 kg N ha^−1^ than at the other N rates (Fig. 6). There was no N rate effect on total extractable soil Mn content in the top 0.9 m (Fig. 6). In Kiboko, Mn uptake (total, grain, and stover) increased as N rate increased (Fig. 6). There was no N rate effect on plant (total, grain, or stover) Mn uptake in Harare (Fig. 6). The extractable soil Mn concentrations in Embu, Kiboko and Harare all decreased as depth increased (Table 3). There was no N rate effect on total extractable soil Mn content in the top 0.9 m in any site (Fig. 6).

**Fig. 7:**
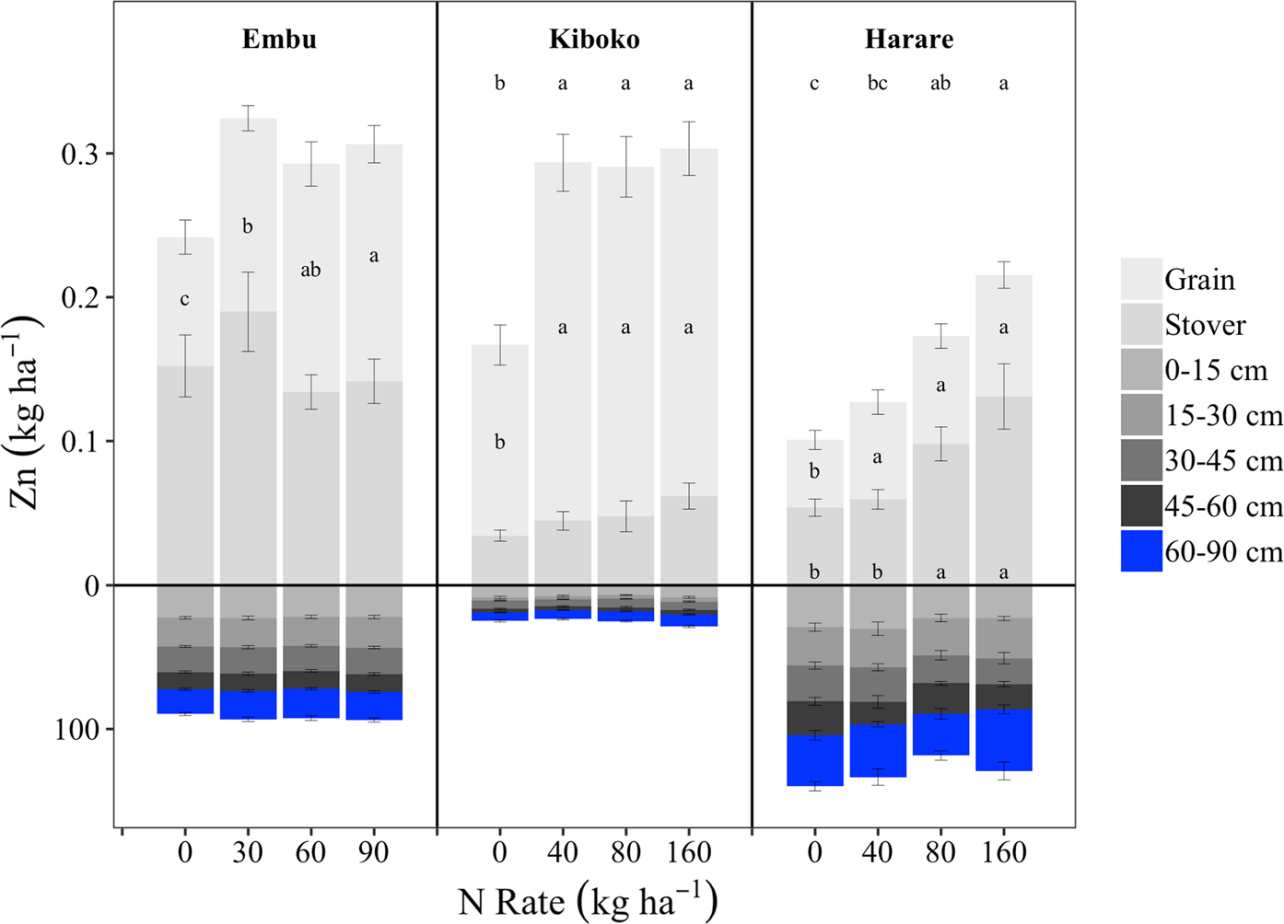
Zinc (Zn) plant-soil balance, averaged across all hybrids, in response to longterm N rates applied to continuous maize at Embu, Kiboko, and Harare. Letters denote differences among the hybrids’ total plant (above bars), grain/stover (on respective parts of bars), or total soil contents (below bars) (*p* ≤ 0.05). Where there are no letters, the difference was not significant

In the top 0.15 m in Embu, extractable soil Zn concentration was high (16 mg kg^−1^ ) (Table 2). In Kiboko, in the top 0.15 m, extractable soil Zn concentration was on the low end of the normal range (3.2 mg kg^−1^) (Table 3). In Kiboko, Embu, there was more Zn content in the grain at 60 and 90 kg N ha^−1^ than at 0 kg N ha^−1^, but there was no N rate effect on total plant or stover Zn contents, grain Zn content was higher at non-zero N rates than at 0 kg N ha^−1^, but did not differ among the non-zero N rates (Fig. 7). In Harare, extractable soil Zn concentration in the top 0.15 m was high (13 mg kg^−1^) (Table 2). Total plant, grain, and stover Zn contents increased as N rate increased (Fig. 7). Extractable soil Zn concentrations generally decreased as depth increased at all three locations (Table 3). There was no significant N rate effect on total extractable soil Zn contents in the top 0.9 m in any site (Fig. 7).

**Fig. 8:**
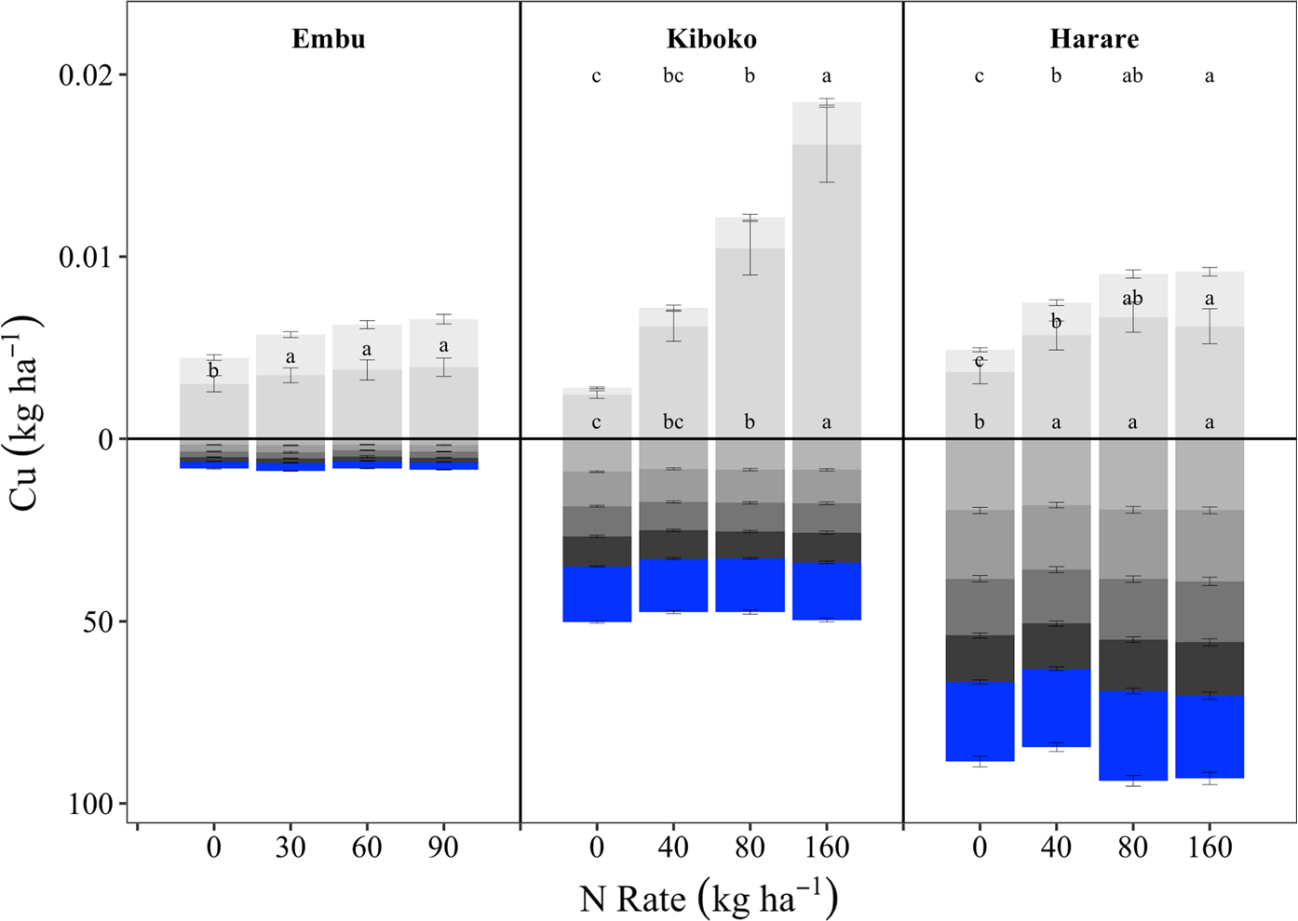
Copper (Cu) plant-soil balance, averaged across all hybrids, in response to long-term N rates applied to continuous maize at Embu, Kiboko, and Harare. Letters denote differences among the hybrids’ total plant (above bars), grain/stover (on respective parts of bars), or total soil contents (below bars) (*p* ≤ 0.05). Where there are no letters, the difference was not significant

In Embu, the extractable soil Cu concentrations in the top 0.15 m were on the low end of normal range (1.0 mg kg^−1^) (Table 3). Grain Cu content was higher at non-zero N rates than at 0 kg N ha^−1^, but did not differ among the non-zero N rates (Fig. 8). In Kiboko total plant, grain, and stover Cu contents increased as N rate increased (Fig. 8). In Harare, the extractable soil Cu concentrations in the top 0.15 m were on the high end of the normal range (8.0 mg kg^−^ ) (Table 2). Total plant, grain, and stover Cu contents were higher at non-zero N rates than at 0 kg N ha^−^, but generally did not differ among the non-zero N rates (Fig. 8). There was no significant N rate effect on total extractable soil Cu contents in the top 0.9 m at any of the three locations (Fig. 8). Extractable soil Cu concentrations decreased as depth increased at each location (Table 3).

### Cumulative effects

The slopes of cumulative grain yield response to change in total soil P as affected by N rate were tested using linear regression analysis. These regressions indicate the potential depletion of the soil P pool at the different sites as the result of higher cumulative grain yields independent of any N rate effect. There was a negative relationship between cumulative yield and inorganic soil P content in Kiboko at 40 kg N ha^−1^ (y = − 0.005x + 25, where y is cumulative grain yield and x is soil total P content; r^2^ = 0.90), but there was no relationship between inorganic soil P and cumulative grain yield at any other N rate or in any other site (data not shown).

**Fig. 9:**
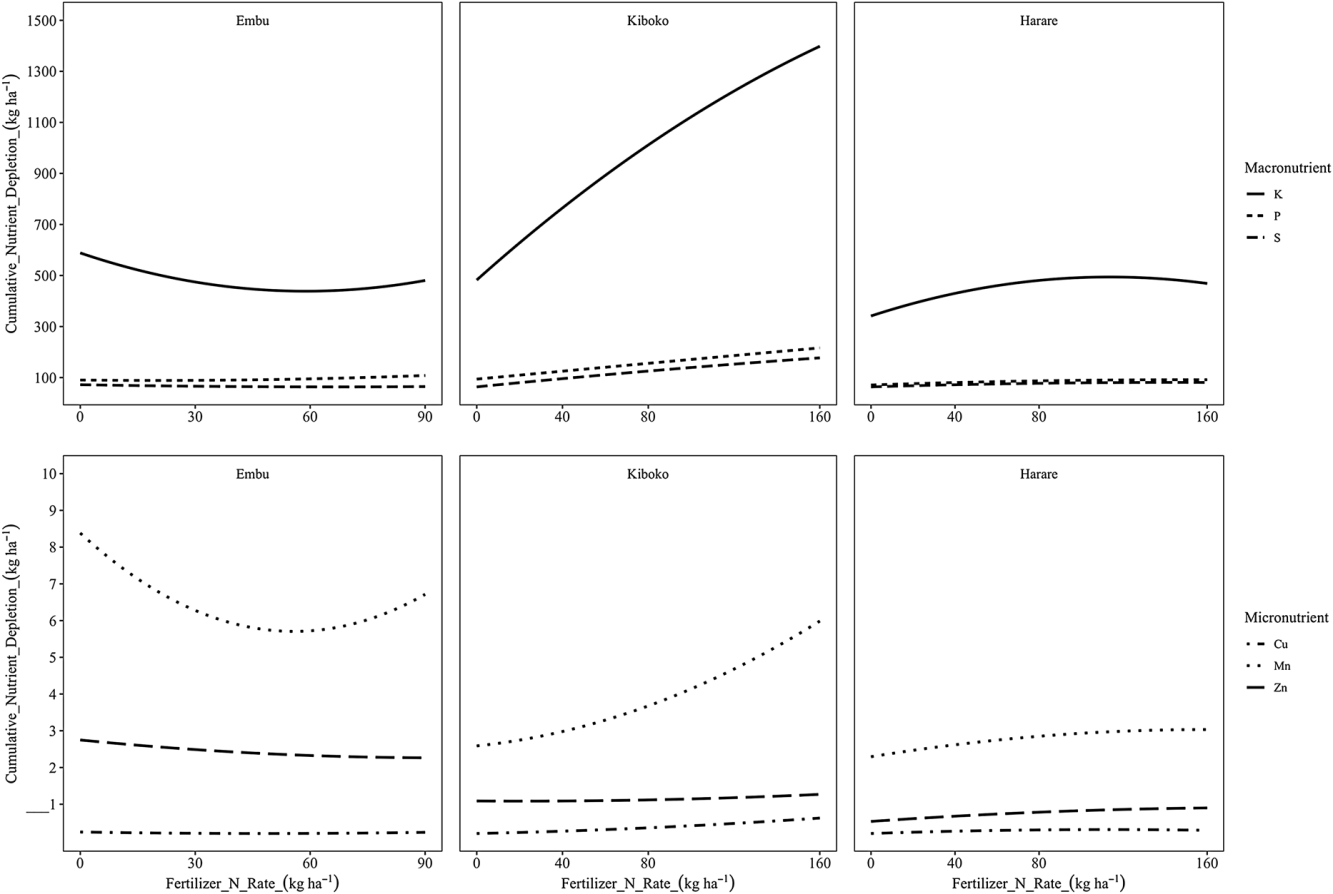
Cumulative nutrient depletion response to N rate averaged across all hybrids at Embu, Kiboko, and Harare

When the cumulative average of total plant nutrient uptake was calculated at each N rate in each site, the cumulative plant removals of P, K, and S increased as N rate increased in all sites (Fig. 9). The cumulative uptake of micronutrients did not change as N rate increased in Embu and Harare (Fig. 9). In Kiboko, the average cumulative nutrient uptake of Mn and Cu also increased as N rate increased, but that of Zn did not change with N rate (Fig. 9). If the calculated AONR within the applied N rate range had been applied each season, 122 kg ha^−1^ P, 468 kg ha^−1^ K, 79 kg ha^−1^ S, 7.7 kg ha^−1^ Mn, 2.9 kg ha^−1^ Zn, and 0.32 kg ha^−1^ Cu would have been cumulatively removed in Embu over 9 seasons, versus 54 kg ha^−1^ P, 374 kg ha^−1^ K, 40 kg ha^−1^ S, 2.2 kg ha^−1^ Mn, 3.9 kg ha^−1^ Zn, and 0.19 kg ha^−1^ Cu in Harare over 5 191 kg ha^−1^ P, 1234 kg ha^−1^ 3.9 kg ha^−1^ Mn, 1.0 kg ha^−1^ Cu in Kiboko over 7 seasons.

## Discussion

### Phosphorus interactions

The high levels of soil acidity and high amorphous Fe levels in Embu and Harare (Table 1) likely resulted in limited soil P availability. This complexing reduced the plant’s access to soil P, resulting in P deficiencies in the hybrids at both sites (Pasley 2018). As a result of 20 kg P ha^−1^ being applied in all three sites and the return of 1/3 of stover residue in Kiboko, the plant-fertilizer P balance was only negative in Kiboko at 80 and 160 kg N ha^−1^ (– 1.1 and – 6 kg P ha^−1^ at 80 and 160 kg N ha^−1^, respectively). If P had not been applied, as is the case in most SSA maize cropping systems, P would have been depleted annually at a rate of7.1–13.2 kgPha^−1^ in Embu, 8.7–12.6 kgPha^−1^ in Harare, and 14.0–39.7 kg P ha^−1^ in Kiboko. Meanwhile, other studies have calculated the average annual P depletion rate in SSA to be 2.5 kg P ha^−1^ (Smaling et al. 1993; Sanchez et al. 1997), confirming that higher grain yields achieved via N fertilizers will result in higher P depletion rates (Tovihoudji et al. 2017). An added complication is that because of high levels of P sorption, previous work on similar soils has found that 110–450 mg P kg^−1^ would need to be added to increase P concentration in the soil solution by 0.1 mg P L^−1^ (Rao et al. 1999).

Soil P was found to be stratified across the rooting zone in all 3 sites, but most significantly in Embu and Harare. The difference in P stratification between Embu/Harare and Kiboko was the result of differences in the soils’ age and parent material. Embu and Harare had older and more weathered soils, resulting in more P depletion and decomposition and, over time, in stratification. Kiboko’s younger soil still has evidence of P sourced from basaltic ash parent material deeper in the rooting profile (Rao et al. 1999; Marques et al. 2004), resulting in a more uniform distribution of P and much higher P levels even at the deepest depth increment sampled. In this study, the stratification of P in Embu and Harare likely limited the plants’ access to P as stress has been found to induce higher angles of steepness in the rooting architecture (He et al. 2003; Trachsel et al. 2013).

Phosphorus was applied as triple superphosphate. Previous studies (Mullins and Sikora 1994; Molina et al. 2009) have found that triple superphosphate can include impurities of Mn, Cu, and Zn. The potential impurities of the triple superphosphate applied in this study were not analyzed, but as the fertilizer was evenly broadcast across all plots, any effect these impurities may have on the results of this study are likely minimal.

### N fertilizer increased nutrient uptake

The application of N increased P uptake, with the exception of a few hybrids in Embu. Feil et al. (1992) and Holou et al. (2011) also found that N fertilizer applications increased P uptake consistently in continuous maize cropping systems. The application of N fertilizer has been found to increase root branching, particularly closer to the surface where P levels are highest (Postma et al. 2014). This increase in root area alone has been found to increase P uptake 5-to 10-fold (Cole et al. 1963; Schenk and Barber 1979; Gahoonia et al. 1997). The application of N fertilizer also increased Zn uptake in Harare where P and Zn were both limiting and soil was compacted, further supporting the hypothesis of a positive root response to N fertilizer.

In the alkaline soil of Kiboko, the application of N also increased P uptake. Unlike in Embu and Harare, however, the increased plant P uptake in Kiboko decreased soil available P, suggesting that the application of N fertilizer enhanced the plant’s ability to take up P in addition to making more P available in the soil. Soil P levels were negatively correlated with soil Zn levels. Previous studies on soils where high P levels limited plant uptake of Zn also found this inverse relationship between P and Zn availability and uptake (Alloway 2009; Gupta et al. 2016).

Soil exchangeable K was low in both Embu and Harare. Like P, K levels were highly stratified across the soil profile in Harare. Coupled with soil compaction, this stratification may have limited maize plant K uptake. Unlike P, however, plant K uptake did not increase as N rate increased in Harare. Further research needs to be conducted to understand the impact of soil K stratification on its availability in tropical soils.

Total plant S content increased as N rate increased in all sites, but only in Harare was there an N rate effect on the soil inorganic S pools. Three major factors can limit S availability for plant uptake: mineralization rate, complexation with other nutrients, and leaching. Although the application of N was found to increase S uptake and, in the case of Harare, potentially S mineralization, this N rate effect has been inconsistent in other field studies (Jamal et al. 2010). High Fe and low pH levels in Embu and Harare coupled with low P levels may have resulted in a strong sorption between Fe and S (Chao et al. 1962).

Plant uptake of Cu increased as N rate increased in both Harare and Kiboko. This N rate effect on Cu uptake was also found by Holou et al. (2011) in Benin. There may have been a link between the increase in S and Cu uptake as soil inorganic S has also been found to complex with Cu (Marschner 2011; Bindraban et al. 2015). The interaction of S with both Fe and Cu may have limited S plant uptake. Soil Cu stratification in all sites was likely linked to that of soil OM which also decreased with depth as Cu solubility, and thus availability, is enhanced by complexing with OM (Pérez-Novo et al. 2008).

The increased uptake of non-N nutrients at higher N rates in this study is evidence of the potential of N fertilizer to enhance plant acquisition of non-N nutrients and, thereby, increase yields (Cakmak 2008). Nevertheless, N fertilizer, even at its highest applied rate in this study did not eliminate non-N nutrient deficiencies. The application of non-N nutrients (in particular, P) in addition to or instead of N can increase yield in SSA to a greater extent than the sole application of N (van der Velde et al. 2013, Tovihoudji et al. 2017).

While the application of N fertilizer may increase both grain yield and non-N nutrient contents in both the grain and stover, the use of N fertilizer to optimize yields in SSA may be a financially unrealistic for most farmers in SSA and have unintended consequences. Each season in this 5-year study, the AONR far exceeded the average N rate applied in SSA (3–5 kg N ha^−1^, Folberth et al. 2013), exceeded the highest applied N rates on multiple occasions, and resulted in the cumulative depletion of soil P, K, and S contents in all three sites. The application of N fertilizer, therefore, may involve risk of perpetuating non-N nutrient depletion that could constrain future maize grain yields.

## Conclusion

Research on nutrient limiting factors in maize systems in SSA has generally focused on low N stress, with relatively little understanding of other limiting nutrient factors and the interactions between and among nutrients. This 5-year study found that while N fertilizer can substantially increase grain yield in these environments, it also enhanced soil depletion of non-N nutrients. Higher N rates increased P, K, and S uptake in all sites irrespective of soil levels, while the response of plant Mn, Zn, and Cu contents to N rate was dependent on interactions with other non-N nutrients (namely P and S) or on plant demand within a season. A N fertilizer-induced increase in the cumulative depletion of Mn and Cu only occurred in Kiboko, where yield response to N was the greatest.

Although we acknowledge the benefits of non-N nutrient additions to improve maize yields, their application rates need to exceed that of micro-dosing to constrain further exacerbation of soil nutrient depletion with the disproportional nutrient uptake of a higher yielding crop (Ibrahim et al. 2015; Tovihoudji et al. 2017). The application of these non-N nutrients at higher levels, however, are not often economically feasible for SSA farmers (Holden 2018). Our study, like others, found evidence that returning crop residue to soil post-harvest can improve yield response to N fertilizer (Shisanya et al. 2009; Vanlauwe et al. 2010, 2015). Improvements to crop residue management may simultaneously improve soil structure and decrease nutrient loss (Bronick and Lal 2005). There is need, therefore, to look at the potential of integrated management approaches to increase immediate yields through the addition of multiple nutrients and then, over time, increase application rates while improving soil structure to combat long-term nutrient depletion.
