# Supplementary material for: Nitrogen fertilizer rate increases plant uptake and soil availability of essential nutrients in continuous maize production in Kenya and Zimbabwe
Source: Nutr Cycl Agroecosyst. 2019 Sep 7;115:373–89. doi: 10.1007/s10705-019-10016-1 (PMC7357727; doi:10.1007/s10705-019-10016-1)
Supplement: Supplementary file 1 [file NCA-2019-s10705-019-10016-1-S1.docx]

*Supplementary Table 1. Rainfall, average temperature for season, maximum daily temperature during critical period (CP), minimum daily temperature during the grain filling period (GF), and daytime and estimated nighttime temperature during the GF for 2013SR-2015SR seasons in Embu, 2012SR-2013SR seasons in Kiboko, 2013/14 and 2014/15 seasons in Harare. “LR” stands for long rains season and “SR” stands for short rains season. Season length is determined as days to black layer which was estimated as twice the number of days from planting to average 50% silking unless crop was harvested prior to this day in which case, the harvest date was used to calculated season length. The CP was defined as the 4 week window around the anthesis-silking interval (ASI), the GF was defined as the time interval between the CP and black layer/harvest. Minimum nighttime temperature during the GF was estimated by the first quartile of the daily temperatures during the GF and the maximum daytime temperature during the GF was estimated by the third quartile of the daily temperatures during the GF.*

|  |  |  | **Rainfall (mm)** | | |  | | | **Temperature ˚C** | | | |
| --- | --- | --- | --- | --- | --- | --- | --- | --- | --- | --- | --- | --- |
| **Site** | **Season** | **Season Length (Days)** | **Season** | **CP** | **GF** | **Average Season** | **CP Max** | **GF Min** | | **GF Q1** | **GF Q3** |  |
| **Embu** | **2013 SR** | 157 | 257 | 57 | 7 | 19.7 | 20.5 | 19.3 | | 20.3 | 21.8 |  |
|  | **2013 LR** | 167 | 128 | 9 | 18 | 19.3 | 20.1 | 16.9 | | 18.3 | 19.9 |  |
|  | **2014 SR** | 153 | 187 | 0 | 74 | 19.8 | 20.7 | 19.0 | | 20.1 | 21.3 |  |
|  | **2014 LR** | 153 | 104 | 20 | 67 | 20.3 | 21.2 | 18.2 | | 19.4 | 20.3 |  |
|  | **2015 SR** | 145 | 132 | 3 | 31 | 20.2 | 20.6 | 19.3 | | 21.0 | 21.9 |  |
| **Kiboko** | **2013 SR** | 159 | 50 | 2 | 41 | 27.9 | 29.6 | 24.9 | | 28.1 | 29.4 |  |
|  | **2013 LR** | 128 | 360 | 7 | 278 | 27.9 | 30.2 | 24.0 | | 25.9 | 28.1 |  |
|  | **2014 SR** | 159 | 53 | 23 | 18 | 27.0 | 28.1 | 26.0 | | 27.5 | 28.8 |  |
|  | **2014 LR** | 128 | 129 | 15 | 85 | 28.4 | 30.4 | 24.6 | | 27.2 | 29.5 |  |
| **Harare** | **2013/14** | 161 | 556 | 64 | 63 | 18.8 | 21.1 | 13.4 | | 15.4 | 18.1 |  |
|  | **2014/15** | 161 | 567 | 56 | 70 | 19.2 | 21.7 | 14.3 | | 16.8 | 18.6 |  |

*Supplementary Table 2. Site management history and details and timeline of data collection.*

| Management | | | | | | | | Timing of Data Collection | | |
| --- | --- | --- | --- | --- | --- | --- | --- | --- | --- | --- |
| Prior Site History | Date Established | N Rate  kg ha^-1^ | N Source | P Rate  kg ha^-1^ | P Source | Population plants ha^-1^ | Row Spacing | Grain Yield | Whole Plant Biomass | For Soil Analysis |
| **Embu** | | | | | | | | | | |
| Continuous maize with no inputs | 2011SR | Seasons 1-4: 0, 40, 80, 120; 5-9: 0, 30, 60, 90 | Calcium Ammonium Nitrate  (26% N) | 20 | Triple Super Phosphate (20% P) | 53,300 | 0.75 m | Every Season | Harvest 2015SR | Post-Harvest 2015SR |
| **Kiboko** | | | | | | | | | | |
| Continuous  sorghum (*Sorghum bicolor L.* ) with no inputs | 2011LR | Seasons 1-2: 0, 40, 80, 120; 3-7: 0, 40, 80, 160 | Calcium Ammonium Nitrate  (26% N) | 20 | Triple Super Phosphate (20% P) | 44,400 | 0.75 m | Every Season | Harvest 2013SR/2014LR | Post-Harvest 2015SR |
| **Harare** | | | | | | | | | | |
| Continous maize with no inputs | 2010/2011 | All Seasons: 0, 40, 80, 160 | Ammonium Nitrate (33.5% N) | 20 | Triple Super Phosphate (20% P) | 66,700 | 0.75 m | Every Season | Harvest 2014/15 | Post-Harvest 2014/15 |

*Supplemental Table 3. Hybrid name, ID, manufacturer, maturity classification (cutoff between Early and Late maturity is 68 days from planting date to anthesis date (AD)), and year of coding (for CIMMYT hybrids)/release (for commercial hybrids) for each site in Embu 2013SR to 2015SR seasons, Kiboko 2012LR to 2014SR seasons, and Harare 2013/14 and 2014/15 seasons.*

| **ID** | **Hybrid Name** | **Manufacturer** | **Maturity Classification** | **Year of Coding/Release** |
| --- | --- | --- | --- | --- |
|  |  |  | **(68 days to AD cutoff between Early and Late)** |  |
| **Embu** | | | | |
| 1 | PHB3253 | DuPont Pioneer | Late | 1996 |
| 2 | DK8031 | Monsanto | Late | 2003 |
| 3 | PAN4M-19 | Pannar Seed | Late | 2008 |
| 4 | Duma43 | Seed Co Ltd | Late | 2004 |
| 5 | H513 | Kenya Seed Co | Late | 1995 |
| 6 | WH403 | Western Seed Co | Late | 2003 |
| **Kiboko** | | | | |
| 1 | WH507 | Western Seed Co | Early | 2006 |
| 2 | CZH0616 | CIMMYT | Early | 2006 |
| 3 | CIM1 | CIMMYT | Early | 2008 |
| 4 | CIM2 | CIMMYT | Early | 2008 |
| 5 | CKH101572 | CIMMYT | Late | 2010 |
| 6 | H513 | Kenya Seed Co | Early | 1995 |
| **Harare** | | | | |
| 1 | CZH132085 | CIMMYT | Late | 2013 |
| 2 | TH127618 | CIMMYT | Late | 2012 |
| 3 | CZL1242 | CIMMYT | Late | 2012 |
| 4 | SC403 | Seed Co Ltd | Early | 1998 |
| 5 | SC513 | Seed Co Ltd | Early | 1997 |
| 6 | PAN413 | Pannar Seed | Late | 1998 |

*Supplementary Table 4. Grain yield, stover biomass dry matter (BM), and stover BM N_Applied_^-1^calculated as grain dry matter / stover biomass dry matter of each hybrid individually and categorically averaged over all N rates in each site in Embu in the 2015SR season, Kiboko in the 2013LR season, and Harare in the 2014/15 season. Which hybrids the ID numbers refer to in each site are specified in Supplementary Table 3. Letters by values denote significant differences among the hybrids, categories, and N rates in a site (p≤0.05). “ns” denotes no significant difference (p>0.05). T*

|  |  | **Embu** | | | **Kiboko** | | | **Harare** | | |
| --- | --- | --- | --- | --- | --- | --- | --- | --- | --- | --- |
| **Factor** | **Hybrid** | **Grain Yield**  **(t ha^-1^)** | **Stover BM**  **(t ha^-1^)** | **Stover BM N_Applied_^-1^ (kg kg^-1^)** | **Grain Yield**  **(t ha^-1^)** | **Stover BM**  **(t ha^-1^)** | **Stover BM N_Applied_^-1^ (kg kg^-1^)** | **Grain Yield**  **(t ha^-1^)** | **Stover BM**  **(t ha^-1^)** | **Stover BM N_Applied_^-1^ (kg kg^-1^)** |
| **Hybrid** | 1 | 5.0 b | 5.9 a | 95 | 3.8 | 7.2 | 93 | 6.2 | 7.7 | 106 |
|  | 2 | 4.1 cd | 6.7 a | 125 | 3.7 | 7.1 | 97 | 5.6 | 6.3 | 87 |
|  | 3 | 4.7 bc | 4.7 b | 92 | 4.0 | 7.2 | 90 | 5.8 | 6.6 | 82 |
|  | 4 | 5.9 a | 4.7 b | 91 | 5.4 | 9.0 | 138 | 5.9 | 6.8 | 87 |
|  | 5 | 4.5 bc | 5.8 a | 110 | 5.5 | 8.8 | 123 | 6.0 | 7.6 | 102 |
|  | 6 | 3.6 d | 5.8 a | 108 | 5.1 | 9.4 | 140 | 6.0 | 6.8 | 91 |
| **Fertilizer N Rate (kg ha^-1^)** | Zero | 3.6 b | 5.1 |  | 1.6 d | 4.3 b |  | 4.0 c | 5.4 b |  |
|  | Low | 4.7 a | 5.9 | 194.2 a | 4.1 c | 8.7 a | 200.7 a | 5.6 b | 7.0 a | 170.3 a |
|  | Medium | 5.2 a | 5.9 | 97.1 b | 5.9 b | 9.8 a | 110.6 b | 6.8 a | 7.9 a | 97.1 b |
|  | High | 5.0 a | 5.4 | 57.5 c | 7.9 a | 10.5 a | 62.5 c | 7.5 a | 7.7 a | 47.3 c |
| **Effect** | **df** | **Level of significance** | | | | | | | | |
| **N** | 3 | <0.0001 | ns | <0.0001 | <0.0001 | <0.0001 | <0.0001 | <0.0001 | <0.001 | <0.0001 |
| **H** | 5 | <0.0001 | <0.0001 | ns | ns | ns | ns | ns | ns | ns |
| **N×H** | 15 | ns | 0.09 | ns | ns | ns | ns | ns | ns | ns |
